# Accuracy of MRI for prediction of response to neo-adjuvant chemotherapy in triple negative breast cancer

**DOI:** 10.1186/1470-7330-15-S1-P18

**Published:** 2015-10-02

**Authors:** GJ Bansal, D Santosh

**Affiliations:** 1 Breast Centre Llandough, University Hospital of Llandough, Penlan Road, Llandough, CF64 2XX, UK

## Aim

The aim of this study was to compare the accuracy of MRI for prediction of response to neo-adjuvant chemotherapy in triple negative breast cancer, with respect to other molecular types.

## Methods

The study comprised of 82 patients who underwent MRI before and after neo-adjuvant chemotherapy but just before surgery. Triple negative cancers were analysed with respect to others subtypes in terms of presentation on MRI (mass or non –mass like enhancement), grade, axillary involvement, shrinkage pattern on MR following chemotherapy and imaging and pathological complete response rate. Accuracy of MRI for prediction of pathological complete response was also compared between different subtypes, by obtaining ROC curves. SPSS (version 21) was used for all data analysis with p value of 0.05 as statistically significant.

## Results

Out of a total of 82 patients, 29 were luminal (HR+/HER -), 23 were triple negative (HR-,HER-), 11 HER positive (HR-,HER+), 19 (HR+/HER+ hybrid). Triple negative cancers are more likely to present as masses on MRI on the pre-chemotherapy MRI scan, were grade 3 and show concentric shrinkage following chemotherapy. Triple negative cancers are more likely to have both imaging and pathological complete response following chemotherapy (p=0.055). For the triple negative group, MR had a sensitivity of 0.745 and specificity of 0.700 (p=0.035), with an area under curve (AUC) of 0.745(95% CI 0.526-0.965).

## Conclusion

Triple negative breast cancers present as masses and show concentric shrinkage following chemotherapy. MRI is most sensitive and specific in predicting response to chemotherapy in this group, compared to others subtypes.

